# Emergence of the super antenna effect in mixed crystals of ytterbium and lutetium complexes showing near-infrared luminescence†

**DOI:** 10.1039/d2ra06007h

**Published:** 2022-10-26

**Authors:** Atsuko Masuya-Suzuki, Satoshi Goto, Rika Nakamura, Ryunosuke Karashimada, Yasuhiro Kubota, Ryo Tsunashima, Nobuhiko Iki

**Affiliations:** Graduate School of Sciences and Technology for Innovation, Yamaguchi University 1677-1 Yoshida Yamaguchi 753-8512 Japan masuya-suzuki@yamaguchi-u.ac.jp; Graduate School of Environmental Studies, Tohoku University 6-6-07 Aramaki-aza Aoba, Aoba-ku Sendai 980-8579 Japan; Department of Chemistry and Biomolecular Science, Faculty of Engineering, Gifu University 1-1 Yanagido Gifu 501-1193 Japan

## Abstract

The synthesis of luminescent molecular crystalline materials requires a good understanding of the luminescence properties of crystals in which many molecules are densely packed. Previously, we studied the near-infrared (NIR) luminescence of a trivalent ytterbium (Yb(iii)) complex with a Schiff base ligand, tris[2-(5-methylsalicylideneimino)ethyl]amine (H_3_L). Herein, we extended our study on the Yb complex (YbL) to enhance and understand its solid-state luminescence *via* mixed crystallization with the lutetium complex (LuL). We prepared (YbL)_*x*_(LuL)_1−*x*_ mixed crystals (*x* = 0.01, 0.05, 0.1, 0.2, 0.3, 0.5, and 0.7) and studied their NIR luminescence properties. The NIR luminescence intensity per Yb(iii) ion for (YbL)_0.01_(LuL)_0.99_ was determined to be two orders of magnitude larger than that for YbL. The excitation spectral shape of (YbL)_0.01_(LuL)_0.99_ was different from the absorption spectral shape of YbL but similar to that of LuL. We attribute this observation to the emergence of an intermolecular energy-migration path. In the mixed crystals, LuL molecules acted as a light-harvesting super antenna for Yb(iii) luminescence. Decay measurements of the NIR luminescence for (YbL)_*x*_(LuL)_1−*x*_ with *x* > 0.2 showed mono-exponential decay, while (YbL)_*x*_(LuL)_1−*x*_ with *x* < 0.1 showed a grow-in component, which reflected the lifetime of the intermediate state for energy migration. The decay lifetime values tended to increase with decreasing *x*, suggesting that Yb(iii) isolation resulted in a reduction in concentration quenching. We propose that the luminescence enhancement in the highly Yb-diluted conditions was mainly caused by an increase in the super antenna effect.

## Abstract 2

In mixed crystals of Yb and Lu complexes, the Lu complex acts as a light-harvesting antenna for the Yb luminescence.

## Graphical abstract

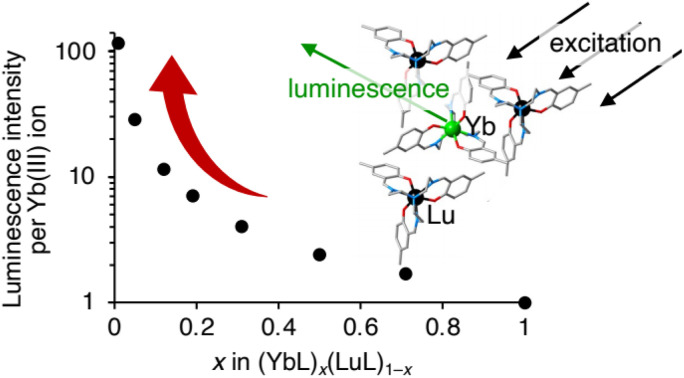

## Introduction

Luminescent molecular crystals have received much attention owing to their fundamental interest and possible applications as color-tunable materials,^1^ sensors,^2^ and optoelectronics.^3^ Understanding how the densely packed molecular conditions in the crystal affect the luminescence property is of central importance for the construction of luminescent materials based on molecular crystals. Trivalent lanthanide (Ln(iii)) complexes with organic ligands are attractive luminescent molecules in which the ligands can act as antennas for the Ln(iii) ions. The energy absorbed by the ligand migrates to the Ln(iii) ion, whose direct excitation is difficult, resulting in the Ln(iii) luminescence.^4^ A large number of luminescent Ln(iii) complexes have been designed by tuning the energy-transfer and relaxation processes. Although some reports have emphasized the effect of the densely packed molecular conditions on Ln(iii) complexes in crystals,^5–9^ the energy transfer and quenching process in crystals remain to be studied in detail.

**Fig. 1:**
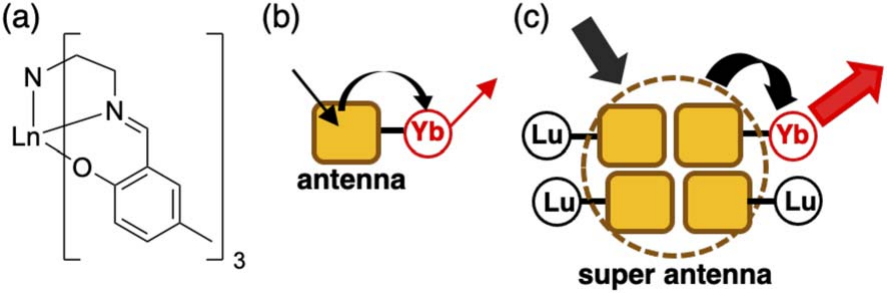
(a) The chemical structure of LnL, and sketches of (b) the antenna effect for a single YbL molecule and (c) the super antenna effect for (YbL)_*x*_(LuL)_1−*x*_.

**Fig. 2:**
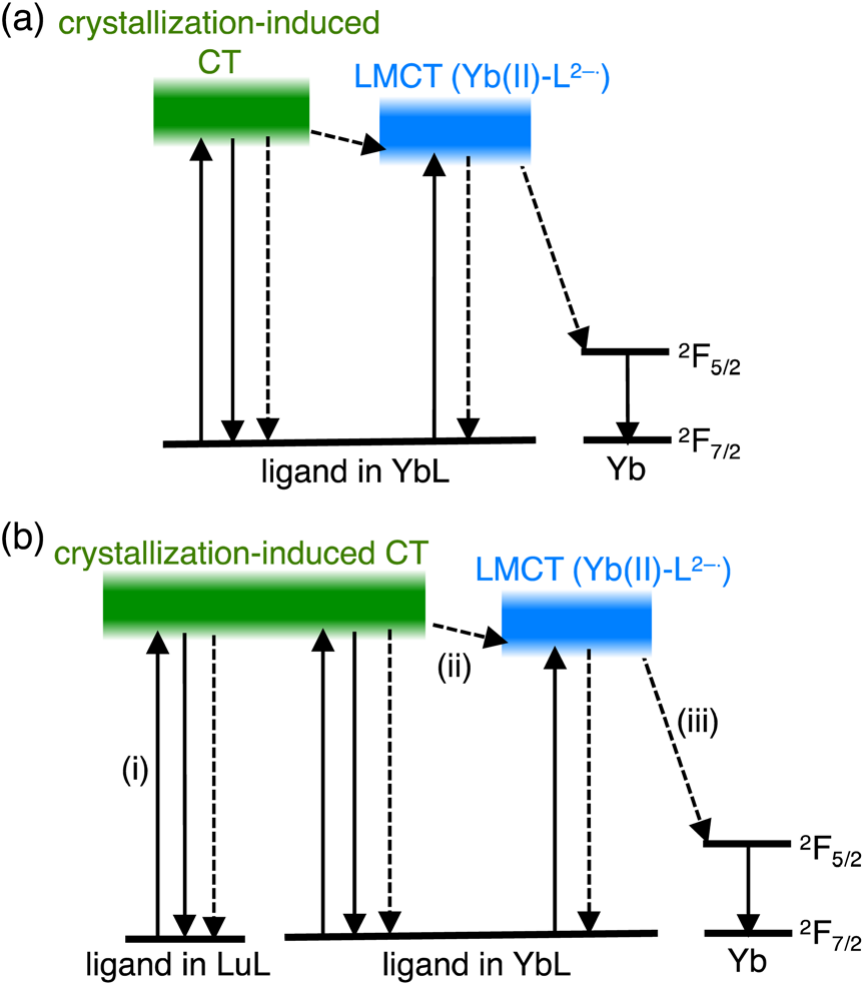
Proposed excitation and relaxation paths for (a) YbL and (b) (YbL)_*x*_(LuL)_1−*x*_.

Recently, we studied a series of Ln(iii) complexes with a tripodal Schiff base, tris[2-(5-methylsalicylideneimino)ethyl]amine (H_3_L), and its derivative.^10–14^ We studied the luminescence properties of the LnL complex with a Schiff base ligand (Fig. 1a, LnL, Ln = Tb, Eu, and Yb) and found that the energy transfer from the ligand to the metal center in TbL and EuL was inefficient, but efficient for YbL. The ligand acted as an antenna for the Yb(iii) luminescence (Fig. 1b) in solution and solid-state.^12^ We showed that conventional energy transfer mechanism through the lowest triplet (T_1_) state of ligands^15^ is not feasible for YbL because of the large energy gap between the T_1_ and Yb(iii) excited states. Instead, we proposed that a redox-mediated mechanism^16^ is operative in YbL. As shown in Fig. 2a, the ligand-to-metal charge transfer (LMCT) state can mediate indirect excitation of the Yb(iii) ion.

In this work, we further extended our study on YbL to enhance and understand its solid-state luminescence *via* mixed crystallization. For the present investigation, we focused on two points from our previous observations.^12^ First, the minimum Yb⋯Yb distance in the crystal structure of YbL was approximately 7 Å (Fig. S1†), which would lead to concentration quenching by intermetallic energy transfer.^8^ Thus, Yb(iii) dilution in the crystals reduces the concentration quenching and enhances the NIR luminescence. To dilute YbL, we prepare mixed crystals of YbL with a trivalent lutetium (Lu(iii)) complex with the tripodal Schiff base (LuL) because of the non-emissive nature of Lu(iii) originating from the filled f-electron configuration (f^14^) and the small difference between the ionic radii of Lu(iii) and Yb(iii) (<0.01 Å),^17^ which allows a homogeneous distribution of the two metal ions. Second, the ligand-centered absorption of the YbL powder was significantly broader than that of the solution. We previously ascribed this broadening to the creation of the excited state involving crystallization-induced charge transfer (CT) from the ligand of one complex to the ligand of the neighboring complex.^12^ From this observation, we hypothesized that LuL in the mixed crystals could act as light-harvesting antennas for the Yb(iii) ion (Fig. 1c). The light absorbed at the ligand in LuL by crystallization-induced CT (Fig. 2b(i)) could be transferred to the LMCT state (Fig. 2b(iii)) and then to the ^2^F_5/2_ level of Yb(iii) (Fig. 2b(iii)). It has been reported that energy transfer from the ligand in the complex of non-emissive Ln(iii) to the ligand in the complex of emissive Ln(iii) results in luminescence enhancement in the suspended solution and micellar solution.^18^ This phenomenon, which is referred to as co-luminescence, can also occur in crystals. However, few studies have examined this phenomenon in crystals in detail. In a few instances, Mikhalyova *et al.* extensively studied the luminescence properties of Tb(iii)/Eu(iii)-doped trivalent gadolinium compounds and called this effect of luminescence enhancement a super antenna effect.^7^ For near-infrared emissive Yb complexes, even fewer studies have been reported on the super antenna effect.

Herein, we prepared a series of (YbL)_*x*_(LuL)_1−*x*_ mixed crystals and studied their NIR luminescence. The luminescence spectral measurements clearly showed that mixed crystallization enhanced NIR luminescence. We examined the contribution of the reduction of the concentration quenching and the increase of the super antenna effect on the luminescence of the mixed crystals using excitation spectral measurements and decay analyses.

## Experimental

### General procedures

Lutetium(iii) trifluoromethanesulfonate [Lu(CF_3_SO_3_)_3_] was purchased from Sigma-Aldrich. Ytterbium(iii) nitrate *n*-hydrate [Yb(NO_3_)_3_·*n*H_2_O] was purchased from Fujifilm Wako Pure Chemical Corp. Lutetium(iii) nitrate tetrahydrate [Lu(NO_3_)_3_·4H_2_O] was purchased from Kanto Chemical Co., Inc. Tris(2-aminoethyl)amine (tren) and 5-methylsalicylaldehyde were purchased from Tokyo Chemical Industry Co. Ltd. Elemental analysis was performed using an NM-10 system (J-Science Lab Co., Ltd). Fast atom bombardment (FAB) mass spectra were obtained using a JEOL JMS-700 mass spectrometer (JEOL Ltd). Inductively coupled plasma atomic emission spectroscopy (ICP-AES) was performed using an iCAP6500 instrument (Thermo Fisher Scientific K.K.). Scanning electron microscopy-energy-dispersive X-ray spectroscopy (SEM-EDX) was conducted using an S-4800 scanning electron microscope (Hitachi High-Tech Corp.) with the EDX analysis system, EMAXEvolution X-max (HORIBA, Ltd). Powder X-ray diffraction (PXRD) data was recorded on a MiniFlex instrument (Rigaku Corp.). The solid samples were gently ground for PXRD measurements using a mortar and pestle.

### Preparation of LuL

The LnL complexes can be obtained by one-pot condensation of tren with 5-methylsalicylaldehyde in the presence of either Ln(NO_3_)_3_ or Ln(CF_3_SO_3_)_3_.^10^ The LuL complex was obtained using a previously reported procedure for the synthesis of YbL,^12^ with Lu(CF_3_SO_3_)_3_ replacing Yb(CF_3_SO_3_)_3_. Yield: 0.143 g (42.6%). Elemental analysis calcd (%) for C_30_H_33_N_4_O_3_Lu: C 53.571, H 4.910, N 8.333; found: C 53.534, H 4.732, N 8.522.

### Preparation of (YbL)_*x*_(LuL)_1−*x*_

A methanol solution (16 mL) containing tren (2.336 g, 8 mmol) was added to methanol solution (104 mL) containing Lu(NO_3_)_3_·4H_2_O (1.732 g, 4 mmol) at 60 °C. The resulting mixture was stirred at 60 °C for 10 min. A methanol solution (16 mL) containing 5-methylsalicylaldehyde (1.680 g, 12 mmol) was added to the reaction mixture and stirred for 5 min at 60 °C. The pale-yellow crude LuL product was collected by filtration, washed with methanol, and dried under reduced pressure. The crude product of YbL was obtained using a procedure similar to that used for LuL, with Yb(NO_3_)_3_·*n*H_2_O in place of Lu(NO_3_)_3_·4H_2_O. The formation of LuL and YbL in each product was confirmed by FAB mass spectral measurements (*m*/*z* = 673 for [Lu(HL)]^+^ and *m*/*z* = 672 for [Yb(HL)]^+^). The mixed crystals (YbL)_*x*_(LuL)_1−*x*_ were obtained from a *N*,*N*-dimethylformamide (DMF) solution containing a stoichiometric amount of the abovementioned crude products. Herein, the preparation of (YbL)_0.5_(LuL)_0.5_ is described as an example. The crude products of YbL (0.200 g) and LuL (0.200 g) were dissolved in 45 mL of DMF at 100 °C. Thirty milliliters of methanol were added to the resulting solution. The solution was allowed to stand overnight at 8 °C, resulting in the precipitation of pale-yellow crystals. The crystals were collected *via* filtration and washed with methanol. After heating the resulting crystals to 40 °C under reduced pressure for 16 h, the mixed crystals were produced. The Yb/Lu ratio in each crystalline sample was determined using ICP-AES measurements. The homogeneous metal distribution and structure of each crystalline sample were determined using SEM-EDX and PXRD, respectively.

### Optical measurement

Solid-state photophysical measurements were performed on the powders that were gently ground using a mortar and pestle. NIR luminescence and excitation spectra were recorded on a Fluorolog-3 spectrometer (HORIBA Jobin Yvon Inc.). The powder samples were placed into a model 1933 solid sample holder (HORIBA Jobin Yvon Inc.). The emitted light was collected using front-face detection. To compare the luminescence intensity, measurements of a series of samples were conducted under identical operating conditions without turning the lamp off. The surface conditions of the solids were made as uniform as possible between the samples to compare the relative luminescence intensities. The reproducibility of the measurements was confirmed. The luminescence intensity values are calculated using the average of at least three independent measurements, with an uncertainty of ±2*σ*. Diffuse reflectance spectra were recorded on a V-570 spectrophotometer (JASCO Corp.) equipped with an ISN-470 integrating sphere (JASCO Corp.). The NIR luminescence lifetime was measured using an LSP-1000 (UNISOKU Co., Ltd) in which a pulsed nitrogen laser (337.1 nm, 3.5 ns) and an InGaAs photodiode were used as the light source and detector, respectively. In this measurement, the NIR luminescence was separated using an 800 nm long-pass filter. The decay profiles were fitted using the nonlinear least-squares method. The lifetime values are calculated as the average of at least three independent measurements, with an uncertainty of ±2*σ*.

## Results and discussion

### Preparation of LuL and (YbL)_*x*_(LuL)_1−*x*_

LuL was prepared using the same procedure as YbL.^12^ The PXRD pattern of LuL (Fig. S2,† blue line) is consistent with the pattern simulated from the single-crystal X-ray structure of YbL (Fig. S2,† brown line),^12^ suggesting that LuL is isostructural with YbL. A series of (YbL)_*x*_(LuL)_1−*x*_ mixed crystals (*x* = 0.01, 0.05, 0.1, 0.3, 0.5, and 0.7) were prepared using a DMF solution containing YbL and LuL in the corresponding mixing ratio. We verified the Yb(iii) fraction of the mixed crystals by ICP-AES measurements of the nitric acid solutions in which the mixed crystals were dissolved. The determined values were in agreement with those expected from the synthetic conditions (Table 1).

**Table 1:** Inductively coupled plasma atomic emission spectroscopy (ICP-AES) and scanning electron microscopy-energy-dispersive X-ray spectroscopy (SEM-EDX) results for (YbL)_*x*_(LuL)_1−*x*_. Experimental values of SEM-EDX are given with an uncertainty of ±2*σ*

*x*	0.01	0.05	0.1	0.2	0.3	0.5	0.7
*x* determined by ICP-AES	0.01	0.05	0.10	0.19	0.31	0.50	0.71
*x* determined by SEM-EDX	0.03(2)	0.07(3)	0.10(2)	0.18(3)	0.32(4)	0.48(2)	0.71(4)

The PXRD patterns of (YbL)_*x*_(LuL)_1−*x*_ (Fig. S2†) were similar to that simulated from the single-crystal X-ray structure of YbL,^12^ indicating that (YbL)_*x*_(LuL)_1−*x*_ was isostructural with YbL. The metal distributions in the crystals were checked by SEM-EDX. EDX mapping (Fig. S3†) confirmed that each metal ion was homogeneously distributed in the mixed crystals. Yb/Lu ratios were estimated using SEM-EDX measurements at three different locations for each sample. As summarized in Table 1, the determined *x* values are in agreement with the global formula of the corresponding sample. Consequently, these results confirm the monophasic nature of the mixed crystals and the random distribution of the metal ions.

### Near-infrared luminescence spectra

**Fig. 3:**
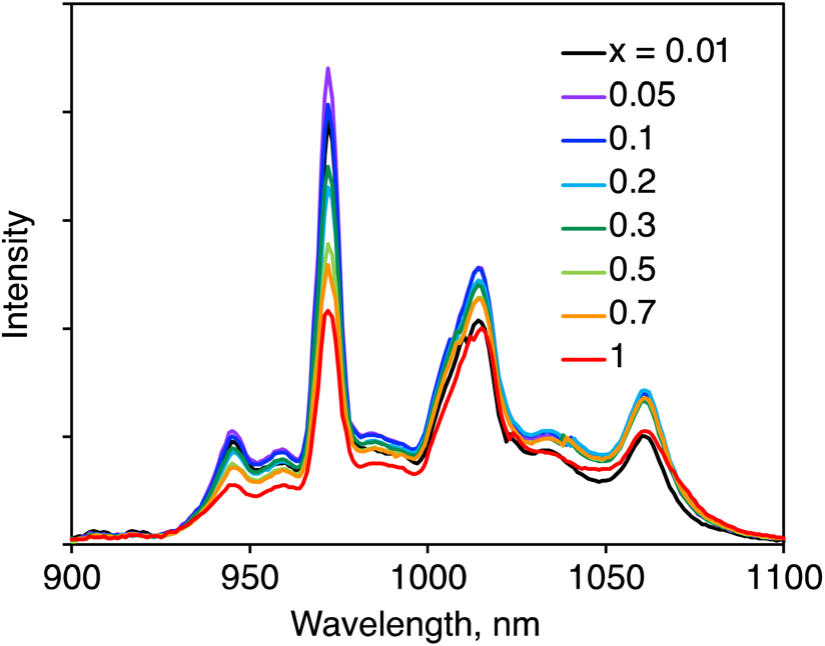
Solid-state near-infrared luminescence spectra of (YbL)_*x*_(LuL)_1−*x*_. *λ*_ex_ = 416 nm.

The NIR luminescence spectra of (YbL)_*x*_(LuL)_1−*x*_ were recorded *via* ligand excitation (Fig. 3). We assigned the NIR luminescence to the ^2^F_5/2_−^2^F_7/2_ transition with Stark splitting, which is related to the Yb(iii) coordination geometry. The peak wavelengths observed for each mixed crystal were nearly identical, indicating that the Stark splitting of Yb(iii) was identical. This means that the coordination environment of Yb(iii) remained unchanged with decreasing *x*.

**Fig. 4:**
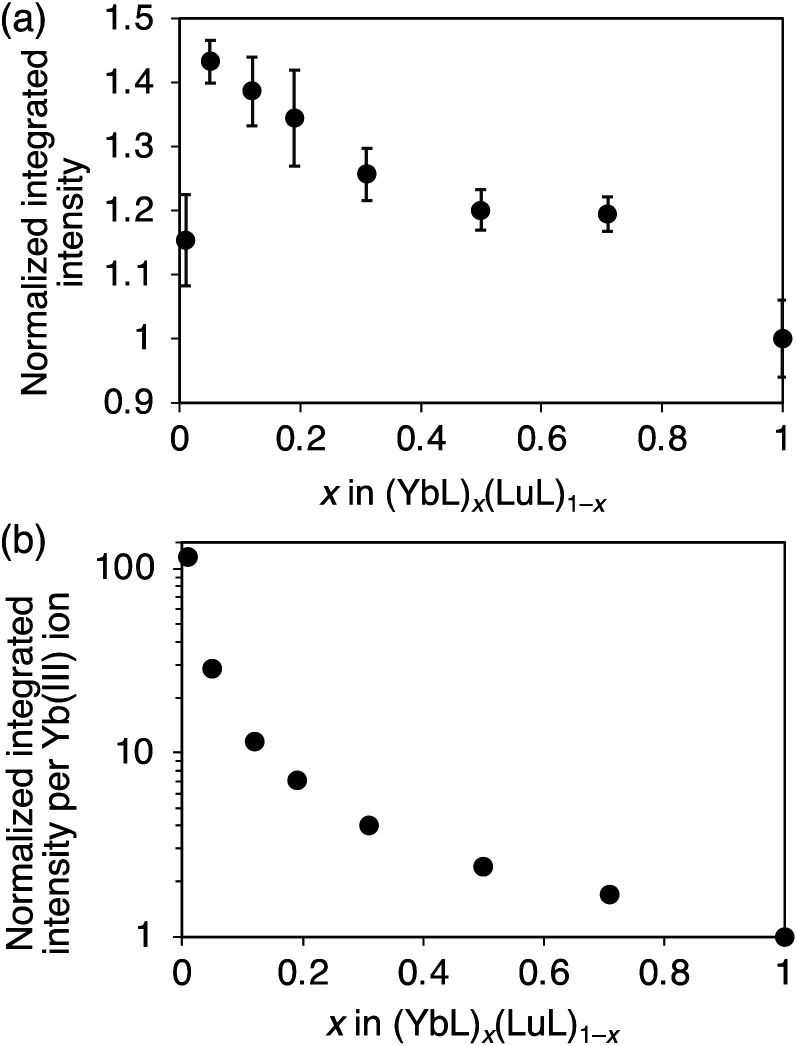
(a) Normalized integrated intensities of the near-infrared luminescence of (YbL)_*x*_(LuL)_1−*x*_ and (b) per Yb(iii) ion of (YbL)_*x*_(LuL)_1−*x*_.

It was essential to compare the luminescence intensities of the powder samples. Generally, the luminescence intensity of a powder sample is influenced by the measurement conditions. To ensure comparative measurement, we ensured that the experimental conditions were maintained as identical as possible, as described in the experimental section. Fig. 4a summarizes the relative integrated luminescence intensity, which is defined as the ratio of the integrated intensity of (YbL)_*x*_(LuL)_1−*x*_ to that of YbL. By decreasing *x* from 1 to 0.01, the intensity increased to a maximum value at *x* = 0.05 before reducing. The appearance of the maximum value at *x* = 0.05 could be explained as the combination of the following two factors: by decreasing *x*, the number of emissive Yb(iii) ions decreases, while the luminescence intensity per Yb(iii) ion increases. The latter factor is clearly seen in Fig. 4b, which shows the luminescence intensity per Yb(iii) ion, defined as the relative luminescence intensity divided by *x*. The value of (YbL)_0.01_(LuL)_0.99_ was found to be two orders of magnitude larger than that of YbL.

**Fig. 5:**
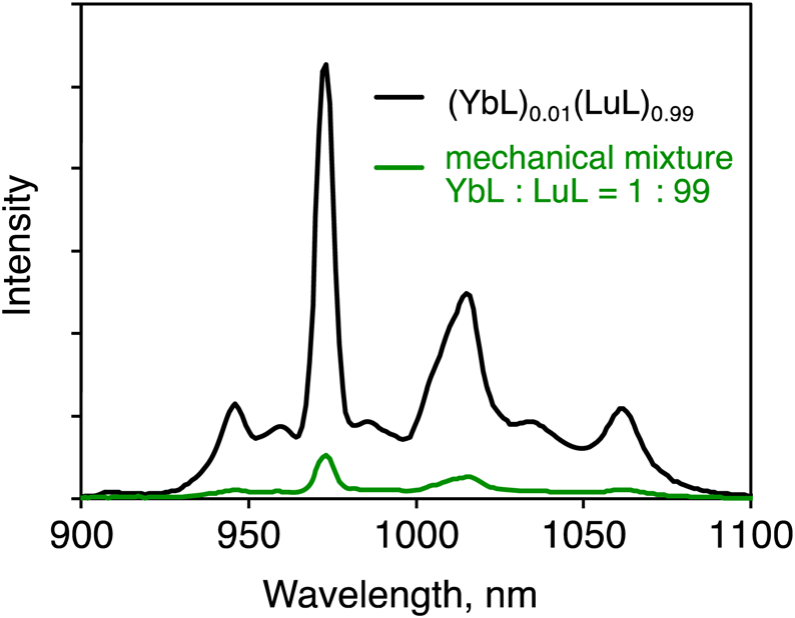
Solid-state near-infrared luminescence spectra of (YbL)_0.01_(LuL)_0.99_ (black line) and a mechanical mixture of YbL and LuL at a 1 : 99 molar ratio (green line). *λ*_ex_ = 416 nm.

To confirm that the luminescence enhancement was caused by the coexistence of YbL and LuL in the same crystal grain, mechanical mixtures of YbL and LuL crystals were prepared for a controlled experiment. A mixture of YbL and LuL at a 1 : 99 molar ratio was gently grounded. Fig. 5 shows the luminescence spectra of this mechanical mixture (green line) and (YbL)_0.01_(LuL)_0.99_ (black line). The luminescence intensity of the mechanical mixture was approximately ten times smaller than that of (YbL)_0.01_(LuL)_0.99_. This result clearly confirms that the luminescence enhancement was caused by the coexistence of YbL and LuL in the same crystal grain.

Furthermore, the comparison of the heights of the emission peaks provides information on reabsorption, which is one of the mechanisms of concentration quenching. In the reabsorption mechanism, light emitted from an emissive center is absorbed by another emissive center. According to a previous report,^9^ the reabsorption of Yb(iii) coordination compounds could be confirmed by the decrease in emission peak heights of the shorter wavelength region of the ^2^F_5/2_−^2^F_7/2_ transition, which overlaps with the ^2^F_7/2_−^2^F_5/2_ absorption. As can be seen in Fig. S4,† the luminescence spectrum normalized at 1060 nm of the mechanical mixture (green line) is very similar to that of (YbL)_0.01_(LuL)_0.99_ (black line). This observation indicates that the degree of reabsorption of the two samples was nearly identical. This result indicates that the reduction in reabsorption is not a significant cause for the luminescence enhancement of (YbL)_0.01_(LuL)_0.99_.

### Excitation spectra

**Fig. 6:**
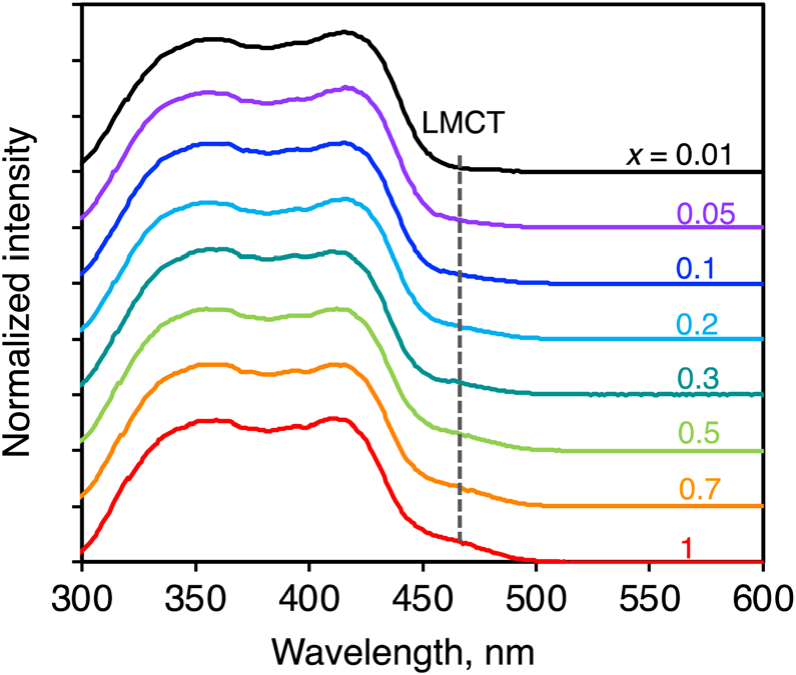
Solid-state excitation spectra of (YbL)_*x*_(LuL)_1−*x*_ normalized at 360 nm. *λ*_em_ = 972 nm.

The information on the ligand-to-Yb(iii) energy migration in (YbL)_*x*_(LuL)_1−*x*_ was obtained from excitation spectral measurements. The spectra were recorded at an emission wavelength of 972 nm. They were mainly composed of ligand-centered excitation bands at approximately 300–450 nm (Fig. 6). For the YbL crystal, we previously assigned the shoulder band on the ligand-centered excitation to LMCT excitation.^12^ As shown in Fig. 6, the relative intensity of the LMCT excitation band to the ligand-centered excitation band reduced as *x* decreased. We examined the absorption properties of the LuL crystal using diffuse reflectance spectroscopy (Fig. S5,† black line). The LuL spectrum consists of ligand-centered absorption without an LMCT shoulder band. This was expected because Lu(iii) is difficult to reduce to Lu(ii). It is important to note that the excitation spectra of (YbL)_*x*_(LuL)_1−*x*_ at low *x* values are different from the absorption of the YbL powder (Fig. S5,† red line), but similar to that of the LuL powder (Fig. S5,† black line). One may notice that the excitation spectra displayed two maxima for YbL (350 and 420 nm), leading to a different shape compared to that of the diffuse reflectance spectra (Fig. S5†). It is possible to understand this by considering that the light at about 400 nm is strongly absorbed at the surface of the sample, resulting in fewer complexes being excited at 400 nm than those excited at 350 or 420 nm. In any case, the excitation spectra of (YbL)_*x*_(LuL)_1−*x*_ showed that a decrease in *x* causes a decrease in the relative intensity of the LMCT excitation band, indicating that the excitation path for Yb(iii) was affected by mixed crystallization.

As a control experiment to confirm the intensity of the LMCT excitation band, we measured the excitation spectrum of a mechanical mixture of YbL and LuL at a 1 : 99 molar ratio (Fig. S6,† green line). In contrast to (YbL)_0.01_(LuL)_0.99_ (Fig. S6,† black line), the mechanical mixture clearly showed the LMCT shoulder band. This result confirms that the presence of LuL molecules around YbL in the crystal structure induces an excitation spectral change. These observations support the hypothesis depicted in Fig. 2b. The excitation spectral shape of (YbL)_*x*_(LuL)_1−*x*_ with lower *x* values could be understood by considering a significant contribution of the absorption at LuL for Yb(iii) excitation.

### Decay analysis

**Fig. 7:**
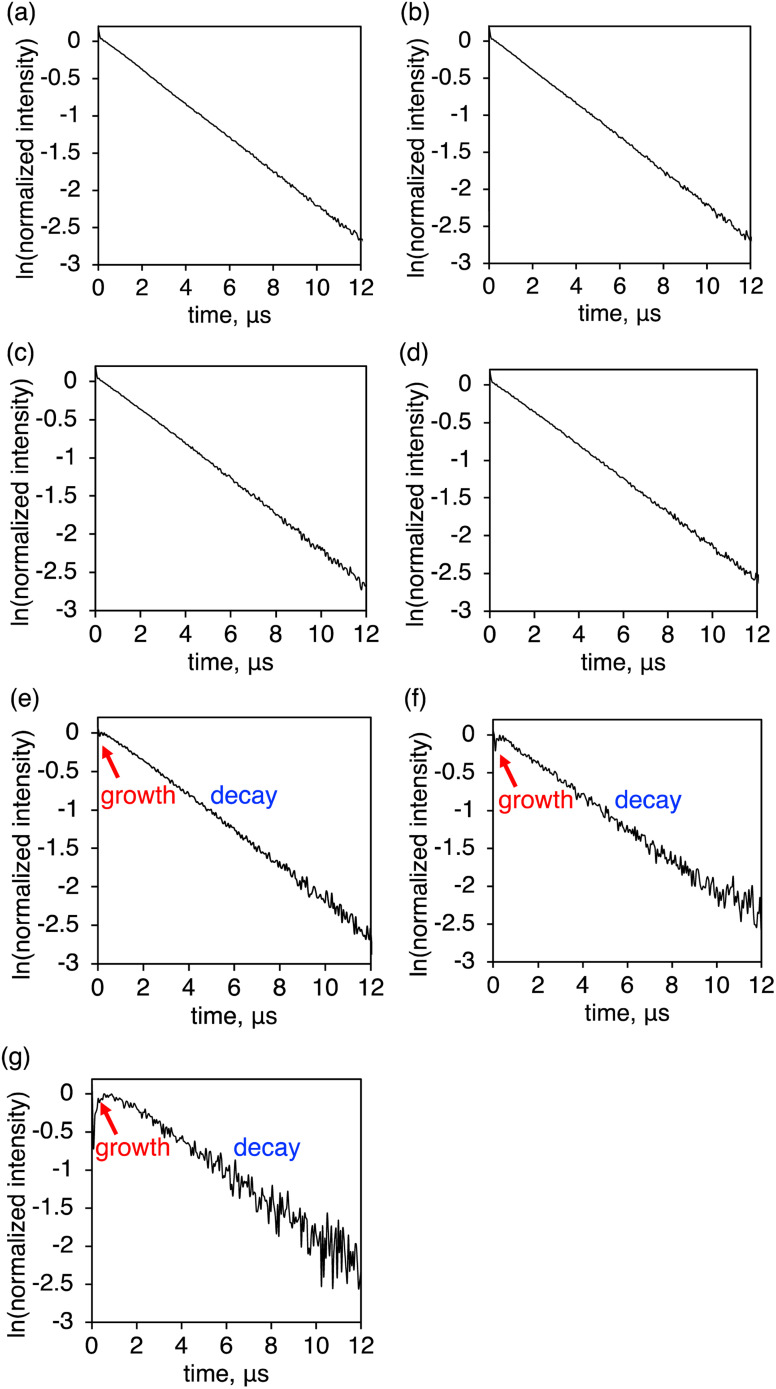
Time profiles of the near-infrared luminescence of (YbL)_*x*_(LuL)_1−*x*_ at (a) *x* = 0.7, (b) 0.5, (c) 0.3, (d) 0.2, (e) 0.1, (f) 0.05, and (g) 0.01. *λ*_ex_ = 337.1 nm.

**Fig. 8:**
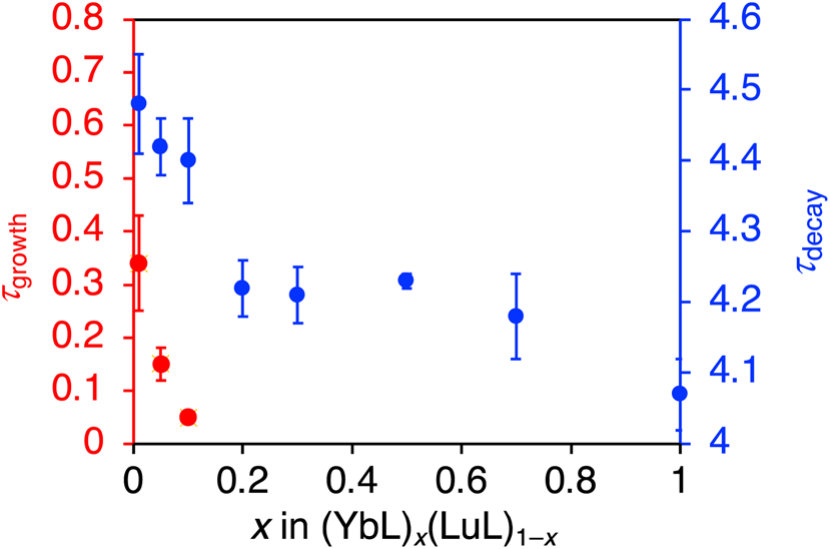
Plots for the growth (*τ*_growth_; red, left axis) and decay (*τ*_decay_; blue, right axis) components of the near-infrared luminescence lifetime against *x* in (YbL)_*x*_(LuL)_1−*x*_. Lifetime values (μs) are shown with an uncertainty of ±2*σ*. *λ*_ex_ = 337.1 nm.

Further insight into the luminescence enhancement was obtained from the time-decay profiles of the NIR luminescence. The mixed crystals with higher YbL fractions (*x* > 0.2) showed single-exponential decay profiles (Fig. 7a–d), while those with lower YbL fractions (*x* < 0.1) showed growth components at the initial part of the profiles (Fig. 7e–g). We presumed that this initial growth component reflected the lifetime of the feeding level to the Yb(iii) excited state (^2^F_5/2_). It has been reported that the presence of an intermediate feeding level can be observed as the initial growth behavior in the time profile when its lifetime is shorter than that of the Ln(iii) excited state.^16*b*,19,20^ We plotted the lifetime values of the growth component (*τ*_growth_) against *x* (Fig. 8, red, left axis). As *x* decreases from 0.1 to 0.01, *τ*_growth_ increased from 0.05(1) to 0.34(9) μs. This observation indicated that the lifetime of the feeding level was elongated by the dilution of YbL. We could not observe growth behavior for a larger value of *x*. This is probably because of the limitation of the time resolution in our measurement system. The crystallization-induced CT and LMCT states might be related to this behavior, but further studies are needed to confirm this assumption.

The plot of the decay component (*τ*_decay_) against *x* is shown in Fig. 8 (blue, right axis). The *τ*_decay_ values tended to increase with decreasing *x*. From the crystal structure of YbL,^12^ quenching by intermetallic energy transfer was predicted as a possible concentration quenching effect. In this crystal structure,^12^ one Yb(iii) ion was surrounded by three Yb(iii) ions with Yb⋯Yb distances of approximately 7 Å in the *bc* plane (Fig. S1a†). This plane-like structure was stacked along the *a*-axis (Fig. S1b†). One Yb(iii) ion was adjacent to two Yb(iii) ions in other planes at a distance of approximately 10 Å. In total, five Yb(iii) ions were located around one Yb(iii) ion with a distance of <10 Å, which is considered to be the minimum distance at which intermetallic energy transfer can occur.^8^ When the mixed crystals with *x* < 1/6 (≈0.17) were exposed to light, the five non-emissive Lu(iii) ions were assumed to exist around a given excited Yb(iii) ion. Intermetallic energy transfer should be suppressed under such conditions. Therefore, we assumed that the apparent increase in *τ*_decay_ observed when *x* decreased from 0.2 to 0.1 (Fig. 8, blue, right axis) corresponded to the suppression of concentration quenching based on intermetallic energy transfer.

In addition to the apparent increase in *τ*_decay_ between *x* = 0.2 and 0.1, a slight increase in *τ*_decay_ was observed in a stepwise manner (Fig. 8, blue, right axis). The precise interpretation of this behavior is not possible in the current study because of the limitation of the uncertainty of our current measurement; therefore, further discussion is needed. Until now, few detailed studies have been reported on the concentration quenching mechanisms in the crystals of Yb(iii) complexes with organic ligands. Recently, Omagari *et al.* discussed the concentration quenching of a Yb(iii) coordination polymer with intermolecular distances > 10 Å in detail, considering several concentration quenching mechanisms. The complicated decay behavior of (YbL)_*x*_(LuL)_1−*x*_ was probably owing to the involvement of these mechanisms, such as quenching by reabsorption, which leads to an increase in the lifetime, and quenching by phonon-assisted energy transfer, which leads to a decrease in the lifetime.

Nevertheless, in view of the purpose of this study, it is important to discuss the major trends in the relation between *τ*_decay_ (Fig. 8, blue) and the luminescence intensity per Yb(iii) ion (Fig. 4b). Notably, the luminescence intensity per Yb(iii) ion rapidly increased at *x* < 0.1, whereas the changes in the *τ*_decay_ were not large. We attribute the main reason for the luminescence enhancement per Yb(iii) ion for the mixed crystals with *x* < 0.1 to the increase of the super antenna effect, rather than the reduction in the concentration quenching.

## Conclusion

The present work demonstrated that the (YbL)_*x*_(LuL)_1−*x*_ mixed crystals display enhanced NIR luminescence. We studied the contribution of the decreased concentration quenching and increased super antenna effect to luminescence enhancement. Under highly diluted conditions (*x* < 0.1), the contribution of the super antenna effect was evidently observed, and the luminescence enhancement was mainly caused by an increase in the super antenna effect rather than a decrease in the concentration quenching. Understanding the nature of this super antenna effect in the crystal may provide a new molecular design strategy for functional materials based on Yb(iii) complexes.

## Conflicts of interest

There are no conflicts to declare.

## Supplementary Material

RA-012-D2RA06007H-s001
