# Supplementary material for: Emergence of the super antenna effect in mixed crystals of ytterbium and lutetium complexes showing near-infrared luminescence
Source: RSC Adv. 2022 Oct 26;12(47):30598–604. doi: 10.1039/d2ra06007h (PMC9597286; doi:10.1039/d2ra06007h)
Supplement: RA-012-D2RA06007H-s001 [file RA-012-D2RA06007H-s001.pdf]

Electronic Supplementary Material (ESI) for RSC Advances.
This journal is © The Royal Society of Chemistry 2022

Supporting Information

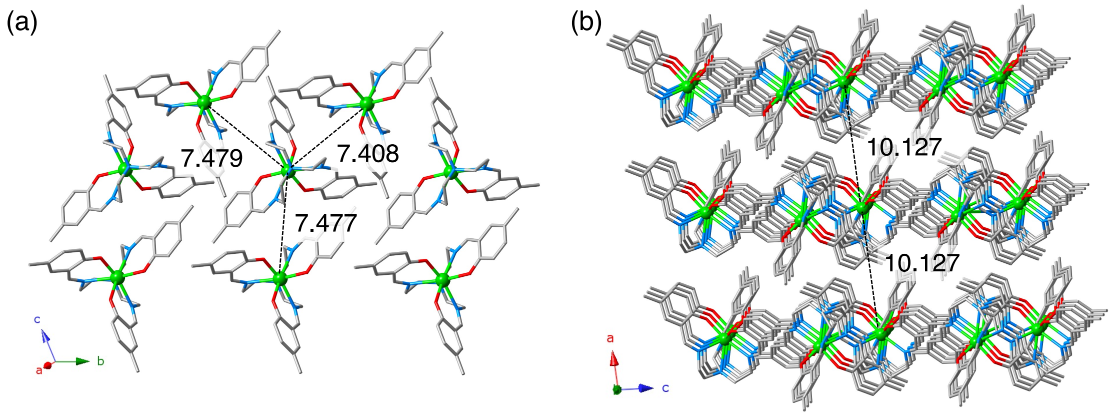

Fig. S1 Crystal packing of YbL reported in *ChemistryOpen*, **2021**, *10*, 46. (a) Plane-like structure in the *bc* plane and (b) stacked plane-like structure along the *a* axis. Gray, blue, and red sticks represent C, N, and O atoms, respectively. Green balls represent Yb(III). Hydrogen atoms were omitted for clarity. Yb···Yb distances below 10 Å are shown.

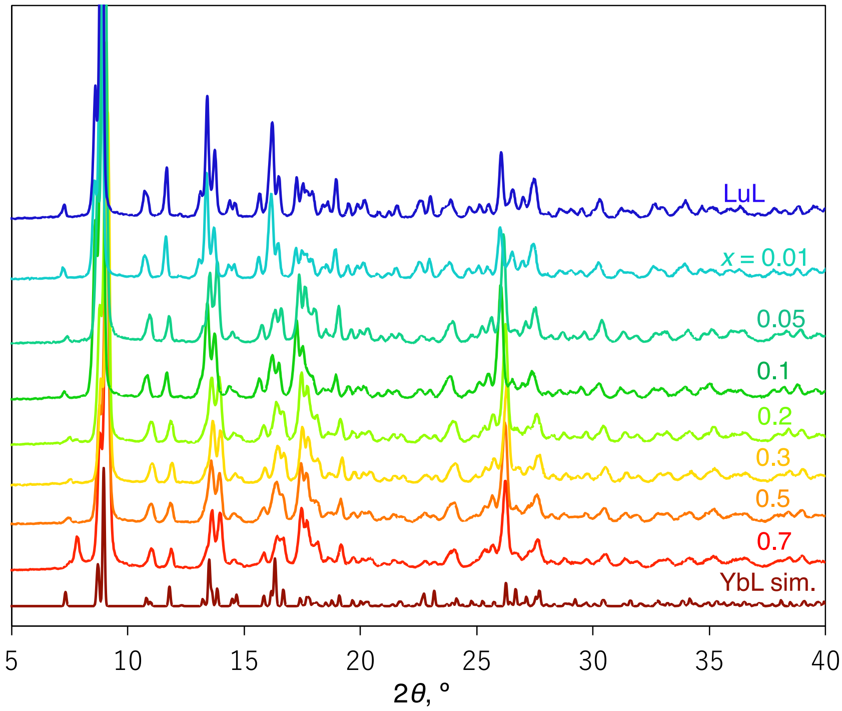

Fig. S2 PXRD patterns of LuL and $(YbL)_x(LuL)_{1-x}$, and that simulated from the single-crystal X-ray diffraction data of YbL, which was reported in *ChemistryOpen*, **2021**, *10*, 46.

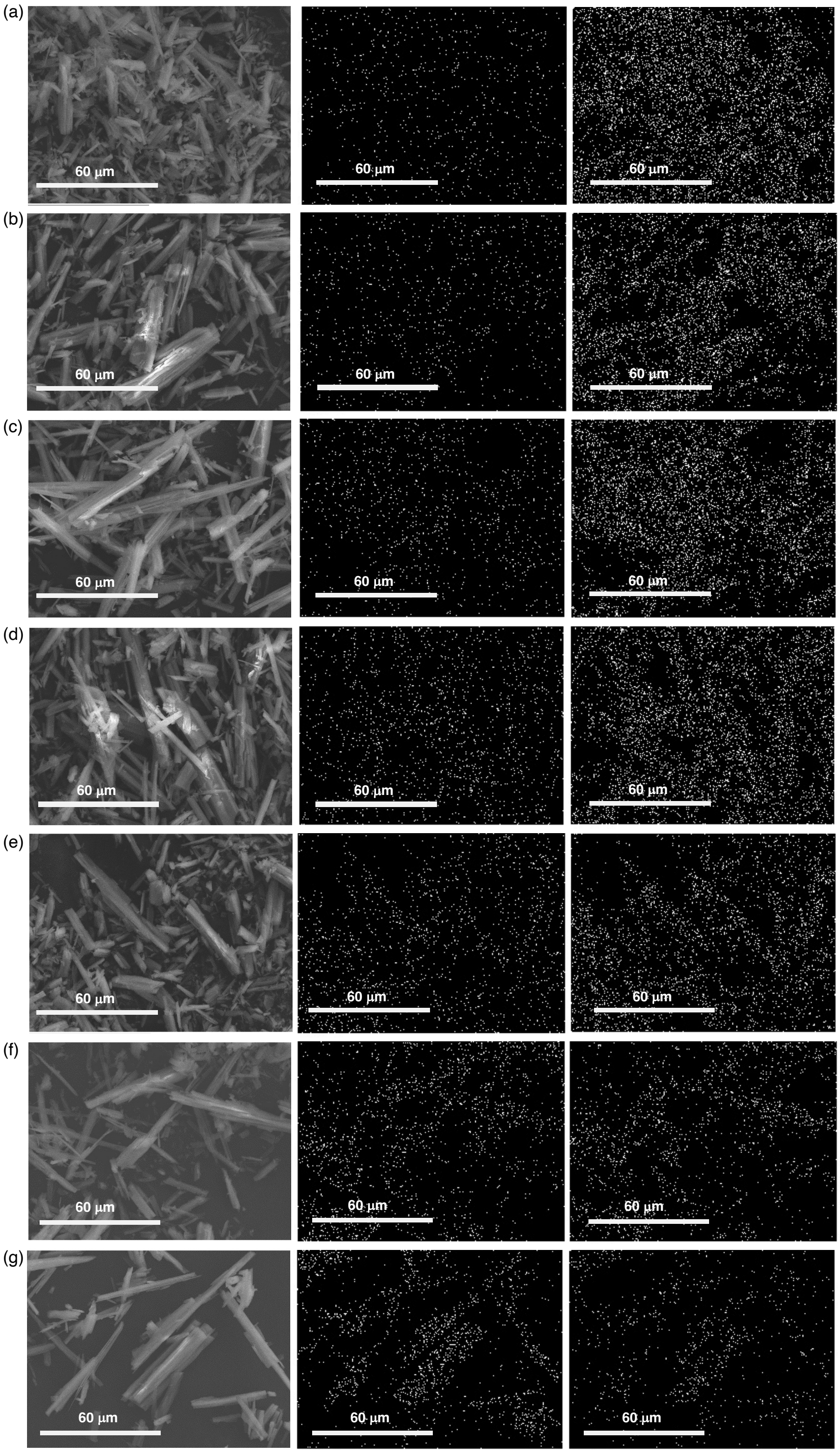

Fig. S3 SEM-EDX image of $(YbL)_x(LuL)_{1-x}$ (x = (a) 0.01, (b) 0.05, (c) 0.1, (d) 0.2, (e) 0.3, (f) 0.5, and (g) 0.7. Left : electron micrograph, middle : EDX map of Yb Lα, right : EDX map of Lu Lα.

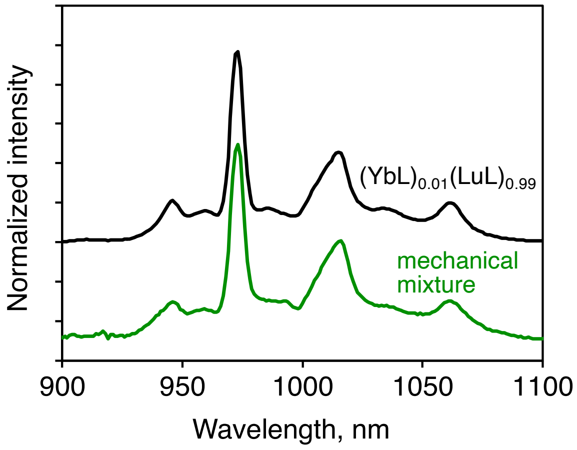

Fig. S4 Solid-state NIR luminescence spectra of $(YbL)_{0.01}(LuL)_{0.99}$ (black line) and mechanical mixture of YbL and LuL at a 1 : 99 molar ratio (green line) normalized at 1061 nm (λ_{ex} = 416 nm).

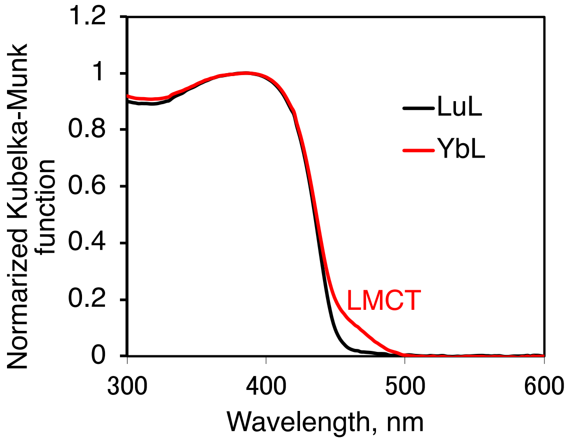

Fig. S5 Diffuse reflectance spectra of the powder of LuL. Red line shows spectrum for YbL reported in *ChemistryOpen*, **2021**, *10*, 46.

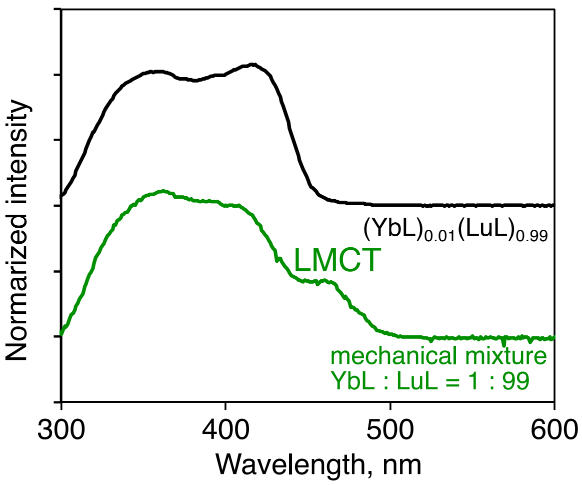

Fig. S6 Solid-state excitation spectra of $(YbL)_{0.01}(LuL)_{0.99}$ (black line) and mechanical mixture of YbL and LuL at a 1 : 99 molar ratio (green line) normalized at 400 nm (λ_{em} = 972 nm).
